# Avoiding “Too Tall” and “Too Short”: The Effect of the Community on the Regulation of Body Height

**DOI:** 10.1002/ajhb.70085

**Published:** 2025-06-12

**Authors:** Michael Hermanussen, Christian Aßmann, Christiane Scheffler

**Affiliations:** ^1^ Pediatrician University of Kiel Kiel Germany; ^2^ Statistics and Econometrics University of Bamberg Bamberg Germany; ^3^ IBB Human Biology University of Potsdam Potsdam Germany

**Keywords:** community effect on height, education, Gini coefficient, gross domestic product, infant mortality

## Abstract

**Background:**

Recent evidence emphasizes the role of social signaling in the regulation of human growth.

**Aim:**

To disentangle the influence of physical living conditions such as wealth, health, nutrition, and education from influences that are transmitted among members of the social group.

We consider the spectrum of all historically possible heights (“transgenerational growth potential”) and disentangle the influence of the physical living conditions from influences that are transmitted among members of the same social group. We ask (1) what is the magnitude of the “transgenerational growth potential”? and (2) to what extent narrows this potential upon entering a specific historic community?

**Samples and Methods:**

We report on the height of more than 14 million German conscripts and recruits born between 1865 and 1975.

**Results:**

Between the late 19th and the late 20th centuries, mean male height increased from 165.8 cm (SD 6.5 cm) to 180.1 cm (SD 7.0 cm). The skewness of the height distribution was always close to zero. Height was statistically associated with living conditions, but the association disappeared when linking characteristics of the within population distribution of height with the disparity of living conditions and economic inequality. Mean height of the social community is a strong attractor of individual height and reduces the “transgenerational growth potential” by more than 50%.

**Conclusion:**

Strong effects of the social community outweigh the effect of individual living conditions and substantially narrow the transgenerational growth potential, to protect against being “too tall” or “too short” within the social community.

## 1 Introduction

This paper makes two important points: 1) body height is a social signal, and 2) the regulation of body height responds to social signals. Responding to social signals is the basis for the fine‐grain regulation of growth and for the comparably small differences in height between people who share the same neighborhood [community effect on height (Aßmann and Hermanussen 2013; Hermanussen et al. 2014)].

It is commonly assumed that adult height is determined by the genetic potential (Deaton 2007; Jelenkovic et al. 2020; Tanner 1986; Visscher et al. 2010; Yengo et al. 2022) with favorable environmental conditions such as wealth (Case and Paxson 2008; Komlos 1994), balanced diet and good health (Perkins et al. 2016) among the factors that allow this potential to be fully realized. But also small family size and a good education (Abuya et al. 2011; Jeong et al. 2018; WHO 2024) have been claimed to contribute to optimum growth. We will adopt this view as the starting point for this study.

We studied the height of German conscripts and recruits born 1865–1975. Most studies on historic trends in adult height refer height to the year of birth (Cole 2000; Hatton and Bray 2010; Hauspie et al. 1997; NCD Risk Factor Collaboration [NCD‐RisC] 2016). We do it differently. As we consider adult height to result from the cumulative effect of all economic, nutritional, social, educational, political, and other factors that are critical to the growth of infants, children, and adolescents, we shift the time scale by 20 years and refer adult height to the historic year at age 20. At this age, most young men have terminated growth. This means cohorts born before the end‐19th century spent all their childhood and adolescence in the politically and economically stable historic period of the German Kaiserreich (1871–1918), whereas cohorts born after 1900 experienced at least parts of their growth in the much more tumultuous years of the First World War and the subsequent time of economic and political disintegration (Glatzer and Glatzer 1983), famine (1916–1919), hyperinflation (1923) and economic failure, and the Great Depression in the late 1920s. Cohorts born during and shortly after the First World War not only suffered from the great famine during early life but also endured political radicalization and, as adolescents, the formation of the Third Reich. Conscripts of the newly formed West German armed forces born shortly before and during the Second World War suffered from infant and early childhood starvation, social rearrangements, foreign occupation, and after 1949, witnessed the formation of the two German countries, the Western Federal Republic of Germany (FRG) and the Eastern Democratic Republic of Germany (GDR). West German cohorts born after the Second World War grew up under stable and much more favorable nutritional circumstances and experienced the so‐called economic miracle (Wirtschaftswunder) and increasing economic prosperity. In 1990, both German countries reunified.

### 1.1 Height and Living Conditions

Data on height and standard deviations (SD) of height were available for German men born between 1865 and 1975. Detailed height data for studying characteristics of the shape and the skewness of the height distributions were available for men born between 1938 and 1975. The total spectrum of body height including all men born between 1865 and 1975 is considered the “transgenerational growth potential” of German men.

In line with current concepts, we started with the assumption that the height of young adults reflects their living conditions (Grasgruber et al. 2016) and referred their height to per capita gross domestic income (GDP), average meat and milk consumption, infant mortality, number of births per woman, and the percentage of young men and women with university qualifications.

As affluent, well‐educated, and well‐nourished populations with low birth rates and low infant mortality rates tend to be tall, we also assumed that the affluent, well‐educated, and well‐nourished people of a given historical period tend to be taller than their poor, ill‐educated, and malnourished contemporaries.

We consider the range of body height of all men born between 1865 and 1975 as a mirror of the ecological tolerance range with regard to the environmental factors that determine the lives of these people. Differences in these factors, that is, income differences, differences in diet, health, family size, and education should be reflected in differences in body height not only between but also within populations.

*We hypothesize:*

1. The width of the height SD at a given historical moment reflects the magnitude of the imbalance between the numbers of affluent and poor people at that moment.
2. The skewness of the body height distribution reflects the degree of social asymmetry. That is, in times when the living conditions such as wealth, health, nutrition, and education were particularly unequally distributed, we expect a greater skewness in the height distribution than in the times when the living conditions were more equally distributed, with similar proportions of affluent and less affluent people.

**FIGURE 1:**
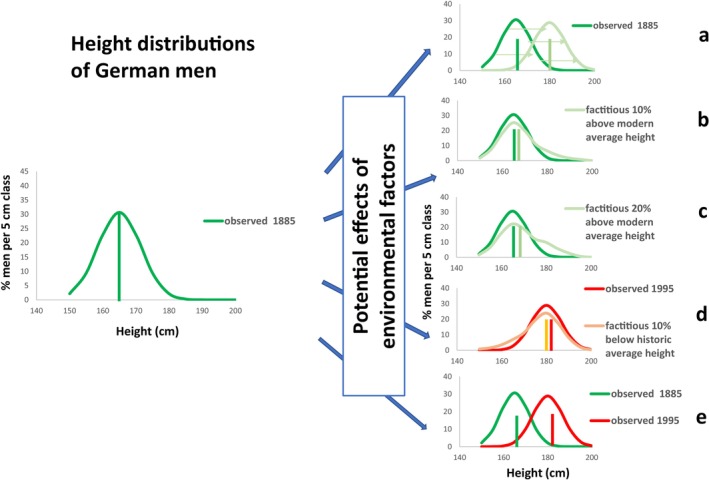
Potential effects of environmental factors on body height. Left: Empirical height distribution of adult German men observed in 1885. Right: Potential height distributions following changes in the environment. Vertical bars indicate mean values of height: (a) Equal changes of environmental factors for everybody yield equal effects on height at the individual level. Mean values for height will change, yet without affecting the standard deviation of height. (b) Factitious improvements of environmental factors for a 10% portion of the 1885 population that allow a few people to grow better and to reach and surpass the average height of their great‐great‐grandchildren 110 years later. The shape of the height distribution should appear distorted; mean value and standard deviation increase. (c) Factitious improvements of environmental factors for a 20% portion of the 1885 population should yield an even greater distortion of the height distribution. (d) Factitious modern environment with a 90% portion of wealthy and tall people and a 10% portion of poor people still below average height of their 1885 ancestors. (e) Empirical late 19th and late 20th centuries height distributions of German men. The figure is indistinguishable from (a).

Changes of environmental factors and living conditions throughout history that differently affect the poor and the affluent social strata should result in concurrent changes of the shape of the height distribution and in consequence, in changes of the mean values of height (Figure 1).

As historical data that would make it possible to directly link height and living conditions at an individual level are not available, we chose an indirect approach and focused on the variation of height and wealth and its associations. We use Gini coefficients for income inequality (Bönke et al. 2015; Gómez León and de Jong 2019) and the household wealth share of the richest top 1% and richest top 10% in the German population (Albers et al. 2020; Bartels and Hesse 2024) to address economic inequality. A Gini coefficient of 0 reflects perfect equality, where everyone has the same income, while a Gini coefficient of 1 reflects maximum inequality, where one person gets everything and the others get nothing.

### 1.2 Social Circumstances and Height

Growth and final height do not only depend on the physical conditions of life. In recent years, social circumstances and particularly the social group itself within which an individual is raised have increasingly gained attention as potential regulators of body height (Hermanussen and Scheffler 2016). Social vertebrates do not only alter behavior in response to changing environmental cues, but can also show adaptive modifications of morphology including growth in size (Buston and Clutton‐Brock 2022). This is called phenotypic plasticity (West‐Eberhard 2003) and refers to the ability of a genotype to express different phenotypes in response to environmental conditions. Social control of size and/or growth has been recognized for at least 50 years. Competing members of the same group adjust in size [strategic growth adjustments (Huchard et al. 2016)] to the size of their competitors. Dominance stimulates growth. Relative size within the group is a social signal.

“Community effects on height” (Aßmann and Hermanussen 2013; Hermanussen et al. 2022) are defined as the result of social interactions within a group, on growth and body height of its members (Hermanussen and Scheffler 2019) and have also been described in human societies (Hermanussen and Scheffler 2016). Community effects on height result in similar height of neighbors, peers and parents, that is, of members of the same social network (Hermanussen et al. 2014).

*We hypothesize:*

3 Absolute height of an individual does not solely reflect its personal state of wealth, health, nutrition, and education, but also mean height of its community. We assume that late 19th century German men were shorter than late 20th century men simply because their 19th century neighbors, peers and parents were shorter

The third hypothesis requires a mental leap: we pool historic and contemporary men, we take the ecological tolerance rate of height, that is, the “transgenerational growth potential”, and consider the “transgenerational” mean and SD for height, which represents all historically possible heights, that is, all heights of millions of men born between 1865 and 1975.

The first two hypotheses presume that the wealthy, healthy, well‐fed, and educated people will predominantly accumulate at the “tall end”, and the disadvantaged and poor at the “short end” of body height distributions. The third hypothesis states that the distance between the two ends of the body height distributions, that is, the distance in height between the wealthy and the poor, is smaller than expected. Community effects serve as attractor for individual height and restrict the full “transgenerational growth potential”. Height within a single annual cohort of conscripts varies significantly less than height of all historic cohorts taken together. We are interested to what extent community effects in height restrict this transgenerational growth potential, and we approach this question by a.

### 1.3 Gedankenexperiment (Thought Experiment)

We consider a case of virtual incarnation: three man can decide when to return to earth and whether to be born in the German Kaiserreich or a century later, in the FRG (see Figure 6). The three men shall be randomly drawn from the “German gene pool” that represents all German men born in all generations between 1865 and 1975. The “German gene pool” encompasses the full, albeit fictitious, spectrum of all historically possible heights that men born from that pool can achieve.

We consider different real‐life communities. In the 1865 community, conditions were such that men born in this year, were able to reach a final height of 165.8 (SD 6.51) cm; they were shortest. In the 1975 community, men were able to reach a final height of 180.1 (SD 6.96) cm; they were tallest when compared with all men born between 1865 and 1975. We are interested to what extent men are attracted by, and adjust to the height of their peers and parents when arriving in their newly chosen community. We ask (1) what is the magnitude of the “transgenerational growth potential”? and (2) to what extent narrows this potential upon entering a specific historic community?

## 2 Samples and Methods

### 2.1 Samples

Height data were obtained from three distinctly different sources.

1. Numbers of German conscripts per centimeter class were available for the East German birth cohorts 1955–1971 (Jäschke, pers. commun.; Jäschke 1991). East German cohorts were mustered at 17 years 9 months, with cohort sizes ranging between 112 304 and 143 105 (altogether 2 171 038). Numbers of West German conscripts per centimeter class were available for the birth cohorts 1938–1975 (Institut für Wehrmedizinalstatistik und Berichtswesen, Remagen 1995). West German cohorts were mustered at 19 to 20 years, with cohort sizes between 439 046 and 140 688 (altogether 12 458 211).
2. Recruit data on mean height and standard deviation of height of the birth cohorts 1900–1919 were obtained from the German Reichswehr (Rass and Rohrkamp 2009). The latter data are smaller in number, with annual cohort sizes ranging between 163 and 832, but they can be considered representative of the male German population (Rass 2023; Rass and Rohrkamp 2009). Reichswehr soldiers were mustered not before age 20 years.
3. Data of persons recruited in the imperial German army who grew up before the First World War under the already affluent conditions of the German Kaiserreich (1871–1918) were obtained from Nowak et al. (2020) with mean height and SD for height of the birth cohorts 1865, 1875, 1885, and 1892. Mean height of Prussian soldiers of the early 19th century was obtained from Schmidt (2000). These soldiers were older than 20 years at the time of measurement.

Except for the East German conscripts, who may not have fully completed growth when drafted (Bogin, Varea, et al. 2018), we considered all other men to have reached final adult height. Comparable sources for data on female height are not available; we therefore limited the study to male height.

### 2.2 Living Conditions

Historic data on (1) per capita gross domestic product (GDP, given in US$) as an indicator of the general economic wealth was obtained from Rahlf (2022); (2) meat consumption as an indirect indicator for food supply from Kanerva (2013) and Teuteberg (1971); (3) milk consumption from Fenton and Owen (1981) and IBISworld (2023) and estimates of milk consumption during the war from Austria (Österreichische Institut für Wirtschaftsforschung 1950) as milk has been shown to be a significant stimulator of child growth (Grenov et al. 2020); (4) infant mortality mainly from statista (Mühlichen et al. 2015; Roesle 1925; Statista 2023) as an indicator of general health; (5) mean number of births per woman as an indicator of family size and an indirect mirror of household economy from Schneider and Dorbritz (2011); and (6) percentage of young men and women with university entrance qualification as an estimate of the general education level of the population was obtained from Müller‐Benedict (2022). We follow the general view and assume that people who enjoy favorable levels of these six indicators will make better use of their genetic height potential than people who suffer from limitations of these indicators.

As up to the end of the 20th century, the proportion of non‐German‐speaking minorities had never risen above 10% (Thiel 2012; Wissenschaftlicher Dienste des Deutschen Bundestages 2009), we considered the “German gene pool” as a stationary factor (Bundeszentrale für politische Bildung 2012) that had not substantially change since 1865.

Gini coefficients for the German Kaiserreich, the Third Reich, and West Germany were obtained from Gómez León and de Jong (2019) and Bönke et al. (2015) and data on household wealth share of the top 1% and top 10% from Albers et al. (2020) and Bartels and Hesse (2024). Reliable data on economic inequality for East Germany have not been found.

As body height distributes close to a Gaussian distribution (Boyd 1980), we plotted the ratio of observed to expected (Gaussian) number of conscripts per centimeter class (o/e‐plots). o/e‐plots are horizontal when the observed data exactly correspond to a Gaussian distribution. o/e‐plots are greater than one when more, and less than one when fewer than expected conscripts are found within a given centimeter class.

### 2.3 Mathematical Considerations

h represents body height. We attempted to find a statistical model that is able to depict the following stylized facts:

For cohort data ${\left\{{\left\{h_{\mathrm{it}}\right\}}_{i=1}^{N_{t}}\right\}}_{i=1}^{T}$, ${\bar{h}}_{t}=\frac{I}{N_{t}}\sum_{i=1}^{N_{t}}h_{\mathrm{it}}$ shows annual increments whereas $S_{h_{t}}^{2}=\frac{I}{N_{t}}\sum_{i=1}^{N_{t}}{\left(h_{\mathrm{it}}-{\bar{h}}_{t}\right)}^{2}$ remains (almost) constant.

The model:

$$h_{\mathrm{it}}\sim p\left(x_{\mathrm{it}}\right)N\left(h_{\mathrm{it}}|\mu_{1}{,\sigma }_{1}^{2}\right)+\left(1-p\left(x_{\mathrm{it}}\right)\right)N\left(h_{\mathrm{it}}|\mu_{2}{,\sigma }_{2}^{2}\right)$$

that is, $h_{\mathrm{ij}}$ obeys a two‐component normal mixed distribution with $x_{\mathrm{it}}$ depicting individual and time specific factors that influence with which probability each of the two factors come into action.

In addition: $\mu_{2}>\mu_{1},\sigma_{1}^{2}>0\;\mathrm{und}\;\sigma_{2}^{2}>0$.

Then, the following applies:

$$E\left[h_{\mathrm{it}}|x_{\mathrm{it}}\right]=p\left(x_{\mathrm{it}}\right)\mu_{1}+\left(1-p\left(x_{\mathrm{it}}\right)\right)\mu_{2}$$

Changes in $x_{\mathrm{it}}$ then immediately go along with changes in ${\bar{h}}_{t}$.

In addition, the following applies:

$$\begin{array}{c} \mathrm{Var}\left[h_{\mathrm{it}}|x_{\mathrm{it}}\right]= \end{array}$$

$$\begin{array}{c} \int h_{\mathrm{it}}-E{\left[h_{\mathrm{it}}|x_{\mathrm{it}}\right]}^{2}\left\{p\left(x_{\mathrm{it}}\right)N\left(h_{\mathrm{it}}|\mu_{1}{,\sigma }_{1}^{2}\right)+\left(1-p\left(x_{\mathrm{it}}\right)\right)N\left(h_{\mathrm{it}}|\mu_{2}{,\sigma }_{2}^{2}\right)\right\}dh_{\mathrm{it}} \end{array}$$

$$\begin{array}{c} =\int \left(h_{\mathrm{it}}^{2}-2h_{\mathrm{it}}E\left[h_{\mathrm{it}}|x_{\mathrm{it}}\right]+E{\left[h_{\mathrm{it}}|x_{\mathrm{it}}\right]}^{2}\right)\\ \;\left\{p\left(x_{\mathrm{it}}\right)N\left(h_{\mathrm{it}}|\mu_{1}{,\sigma }_{1}^{2}\right)+\left(1-p\left(x_{\mathrm{it}}\right)\right)N\left(h_{\mathrm{it}}|\mu_{2}{,\sigma }_{2}^{2}\right)\right\}dh_{\mathrm{it}} \end{array}$$

$$\begin{array}{c} =p\left(x_{\mathrm{it}}\right)\left[\sigma_{1}^{2}+\mu_{1}^{2}-2\mu_{1}E\left[h_{\mathrm{it}}|x_{\mathrm{it}}\right]+E{\left[h_{\mathrm{it}}|x_{\mathrm{it}}\right]}^{2}\right]\\ +\left(1-p\left(x_{\mathrm{it}}\right)\right)\left[\sigma_{2}^{2}+\mu_{2}^{2}-2\mu_{2}E\left[h_{\mathrm{it}}|x_{\mathrm{it}}\right]+E{\left[h_{\mathrm{it}}|x_{\mathrm{it}}\right]}^{2}\right]\\ =p\left(x_{\mathrm{it}}\right)\left[\sigma_{1}^{2}+\mu_{1}^{2}-2\mu_{1}E\left[h_{\mathrm{it}}|x_{\mathrm{it}}\right]+E\left[h_{\mathrm{it}}|x_{\mathrm{it}}\right]\right] \end{array}$$

$$\begin{array}{c} +\left(1-p\left(x_{\mathrm{it}}\right)\right)\left[\sigma_{2}^{2}+\mu_{2}^{2}-2\mu_{2}E\left[h_{\mathrm{it}}|x_{\mathrm{it}}\right]\right]+E{\left[h_{\mathrm{it}}|x_{\mathrm{it}}\right]}^{2} \end{array}$$

$$\begin{array}{c} =p\left(x_{\mathrm{it}}\right)\left[\sigma_{1}^{2}+\mu_{1}^{2}-2\mu_{1}\left(p\left(x_{\mathrm{it}}\right)\mu_{1}+\left(1-p\left(x_{\mathrm{it}}\right)\right)\mu_{2}\right)\right]+\\ +\left(1-p\left(x_{\mathrm{it}}\right)\right)\;\left[\sigma_{2}^{2}+\mu_{2}^{2}-2\mu_{2}\left(p\left(x_{\mathrm{it}}\right)\mu_{1}+\left(1-p\left(x_{\mathrm{it}}\right)\right)\mu_{2}\right)\right] \end{array}$$

$$\begin{array}{c} +p{\left(x_{\mathrm{it}}\right)}^{2}\mu_{1}^{2}+(1-p{\left(x_{\mathrm{it}})\right)}^{2}\mu_{2}^{2}+2\mu_{1}\mu_{2}p\left(x_{\mathrm{it}}\right)\left(1-p\left(x_{\mathrm{it}}\right)\right) \end{array}$$

$$\begin{array}{c} =p\left(x_{\mathrm{it}}\right)\sigma_{1}^{2}+p\left(x_{\mathrm{it}}\right)\mu_{1}^{2}-2\mu_{1}^{2}p{\left(x_{\mathrm{it}}\right)}^{2}-2\mu_{1}\mu_{2}p\left(x_{\mathrm{it}}\right)\left(1-p\left(x_{\mathrm{it}}\right)\right)\\ +\left(1-p\left(x_{\mathrm{it}}\right)\right)\sigma_{2}^{2}+\left(1-p\left(x_{\mathrm{it}}\right)\right)\mu_{2}^{2}-2\mu_{1}\mu_{2}p\left(x_{\mathrm{it}}\right)\left(1-p\left(x_{\mathrm{it}}\right)\right)-2\mu_{2}^{2}(1-p{\left(x_{\mathrm{it}})\right)}^{2}\\ +p{\left(x_{\mathrm{it}}\right)}^{2}\mu_{1}^{2}+(1-p{\left(x_{\mathrm{it}})\right)}^{2}\mu_{2}^{2}+2\mu_{1}\mu_{2}p\left(x_{\mathrm{it}}\right)\left(1-p\left(x_{\mathrm{it}}\right)\right) \end{array}$$

$$\begin{array}{c} =p\left(x_{\mathrm{it}}\right)\sigma_{1}^{2}+\left(1-p\left(x_{\mathrm{it}}\right)\right)\sigma_{2}^{2}-2\mu_{1}\mu_{2}p\left(x_{\mathrm{it}}\right)\left(1-p\left(x_{\mathrm{it}}\right)\right)\\ -\left(1-p\left(x_{\mathrm{it}}\right)\right)\sigma_{2}^{2}+\left(1-p\left(x_{\mathrm{it}}\right)\right)\mu_{2}^{2}-2\mu_{1}\mu_{2}p\left(x_{\mathrm{it}}\right)\left(1-p\left(x_{\mathrm{it}}\right)\right)-2\mu_{2}^{2}(1-p{\left(x_{\mathrm{it}})\right)}^{2} \end{array}$$

$$\begin{array}{c} -p{\left(x_{\mathrm{it}}\right)}^{2}\mu_{1}^{2}-(1-p{\left(x_{\mathrm{it}})\right)}^{2}\mu_{2}^{2}+p\left(x_{\mathrm{it}}\right)\mu_{1}^{2}+p\left(x_{\mathrm{it}}\right)\mu_{2}^{2} \end{array}$$

$$\begin{array}{c} -1+2\;p\left(x_{\mathrm{it}}\right)-p{\left(x_{\mathrm{it}}\right)}^{2}+\left(1-p\left(x_{\mathrm{it}}\right)\right) \end{array}$$

$$\begin{array}{c} =p\left(x_{\mathrm{it}}\right)\sigma_{1}^{2}+\left(1-p\left(x_{\mathrm{it}}\right)\right)\sigma_{2}^{2}-2\mu_{1}\mu_{2}p\left(x_{\mathrm{it}}\right)\left(1-p\left(x_{\mathrm{it}}\right)\right) \end{array}$$

$$\begin{array}{c} +p\left(x_{\mathrm{it}}\right)\left(1-p\left(x_{\mathrm{it}}\right)\right)\mu_{1}^{2}+p\left(x_{\mathrm{it}}\right)\left(1-p\left(x_{\mathrm{it}}\right)\right)\mu_{2}^{2} \end{array}$$

With

$\sigma_{1}=\sigma_{2}$ and p=0.5

$$\text{Variance}=\sigma^{2}-2\mu_{1}\mu_{2}\frac{1}{4}+\frac{1}{4}\mu_{1}^{2}+\frac{1}{4}\mu_{2}^{2}$$

$$\text{Variance}=\sigma^{2}+\frac{1}{4}{\left(\mu_{1}-\mu_{2}\right)}^{2}$$

The calculation shows that in the moment where both components are equal, $p\;\epsilon \left(0,1\right)$, the variance grows proportionally to ${\left(\mu_{1}-\mu_{2}\right)}^{2}$ until p=0.5. This means that with increasing average body height the height SD first increases and thereafter decreases again.

Correlation analyses, simulation, and graphics were handled with the programming language R. R is Free Software under the terms of the Free Software Foundation's GNU General Public License in source code from the R Foundation (R Development Core Team 2024).

## 3 Results

We describe historic trends in living conditions, economic inequality and body height between 1885 and 1995. Thereafter, we develop an estimate of the “transgenerational growth potential” and present the outcome of the thought experiment.

### 3.1 Trends in the General Living Conditions

Except for milk consumption that fluctuated around 100 kg per person with no discernible trend, all living conditions improved over time.

The per capita gross domestic product (GDP) increased from around 2000 international dollars (GK$) at the beginning of the German Kaiserreich in 1871 to about 3500 GK$ at the beginning of the First World War. The GDP reached some 5000 GK$ at the beginning of the Second World War; it sharply decreased thereafter but quickly recovered in West Germany, and already in the mid‐1950s reached the pre‐war level and continued to increase up to 15,000 GK$ in 1995.

The annual meat consumption varied around 45 kg per capita before 1914, yet, with major differences among the social strata as exemplified by Teuteberg (1971): “a detailed analysis of the eating of beef and pork between 1867 and 1875 in twenty four towns in Saxony, each with more than 8000 inhabitants, shows that the per capita consumption ranged from 17.5 kg (Schneeberg) and 28 kg (Zwickau) to 73.6 kg (Leipzig)”. The meat consumption reached maxima in the mid‐1990s with almost 100 kg per capita.

Infant mortality rapidly declined from 225 permille at the beginning of the 20th century to 6 permille in 1995; the mean number of births per women dropped from 4.7 children in 1900 to 1.2 in 1995.

**FIGURE 2:**
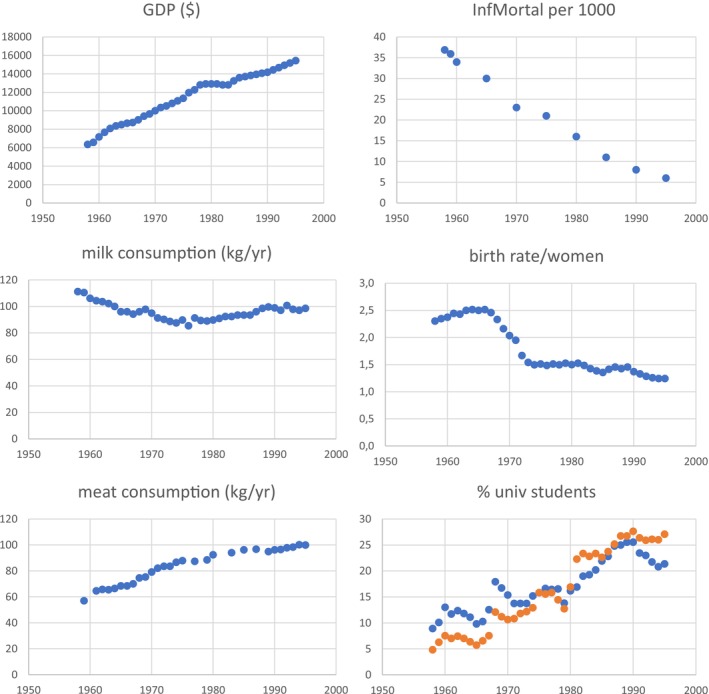
Trends after the Second World War in gross domestic product (GDP) in international dollars, per capita milk and meat consumption in kg/year, infant mortality (InfMortal) per 1000 births, birth rate per women, and percentage of young men and women with university entrance qualification (orange: female; blue: male) in the FRG.

The improvements in living conditions were particularly stable after the Second World War. Figure 2 illustrates these trends, Table 1 summarizes the multiple correlations between height and economic variables in West German society (birth cohorts 1938–1975).

**TABLE 1:** Multiple correlations between body height, height SD, gross domestic product (GDP) in international dollars, per capita meat and milk consumption in kg/year, infant mortality (InfMortal) per 1000 births, birth rate per women, and percentage of young men and women with university entrance qualification between 1958 and 1995 (birth cohorts 1938–1975) in the FRG.

	1958–1995	Height	Height SD	GDP	Meat	Milk	InfMort	Birth rate	% male students
Height	**0.99**								
Height SD	**0.96**	**0.95**							
GDP	**0.99**	**0.99**	**0.93**						
Meat	**0.96**	**0.97**	**0.89**	**0.98**					
Milk	**−**0.34	**−**0.38	**−**0.14	**−**0.45	**−**0.42				
InfMort	**−0.99**	**−0.99**	**−0.93**	**−0.99**	**−0.97**	0.64			
Birth rate	**−0.91**	**−0.93**	**−0.85**	**−0.93**	**−0.96**	0.5	**0.94**		
% male students	**0.9**	**0.9**	**0.86**	**0.87**	**0.86**	**−**0.17	**−0.94**	**−0.79**	
% female students	**0.97**	**0.97**	**0.95**	**0.94**	**0.93**	**−**0.23	**−0.97**	**−0.88**	**0.95**

*Note:* Bold numbers indicate significant correlations (blue—positive; red—negative).

### 3.2 Trends in Economic Inequality

**FIGURE 3:**
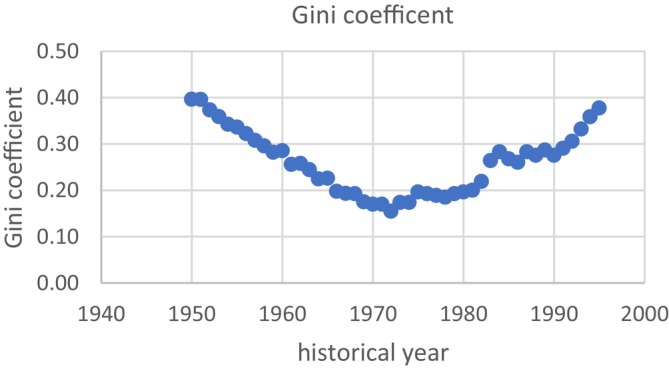
Trend in income inequality (Gini coefficients) in the West Germany population after the Second World War. A Gini coefficient of 0 reflects perfect equality, where everyone has the same income, while a Gini coefficient of 1 reflects maximum inequality.

In contrast to the rather uniform improvements in living conditions, the changes in social and economic equality fluctuated. The Gini coefficient in West Germany sharply decreased from 0.4 at the end of the Second World War to a minimum of 0.15 in 1972, and thereafter rose again to 0.38 in 1995 (Figure 3). Data on household wealth share of the richest top 1% and top 10% were less plentiful, but still showed substantial decreases from 34% (top 1%) and 70% (top 10%) in the mid‐1930s to 25% (top 1%) and 50% (top 10%) in the mid‐1970s (Albers et al. 2020; Bartels and Hesse 2024).

#### 3.2.1 General Trends in Height and Height Variation in Germany

At the beginning of the 19th century, the mean adult height of Prussian soldiers was 163 cm (Schmidt 2000). Adult height slightly increased in the following years but remained comparably short. Between 1885 and the beginning of the First World War (birth cohorts 1865–1894), the mean height of German men increased from 165.8 to 166.8 cm, and thereafter by 13.3 to 180.1 cm (birth cohort 1975). The skewness of the height distribution in German men born between 1938 and 1975 remained close to zero, with no apparent trend. The SD for height decreased from 6.51 cm in the birth cohort 1865 to little more than 6.0 cm in men born in the early 20th century and thereafter increased to 6.96 cm in the birth cohort 1975 (Table S1). The height data emphasize that the changes in income disparity are not reflected in the distribution of height (Table S1).

## 4 Post‐World War 2 Trends

**FIGURE 4:**
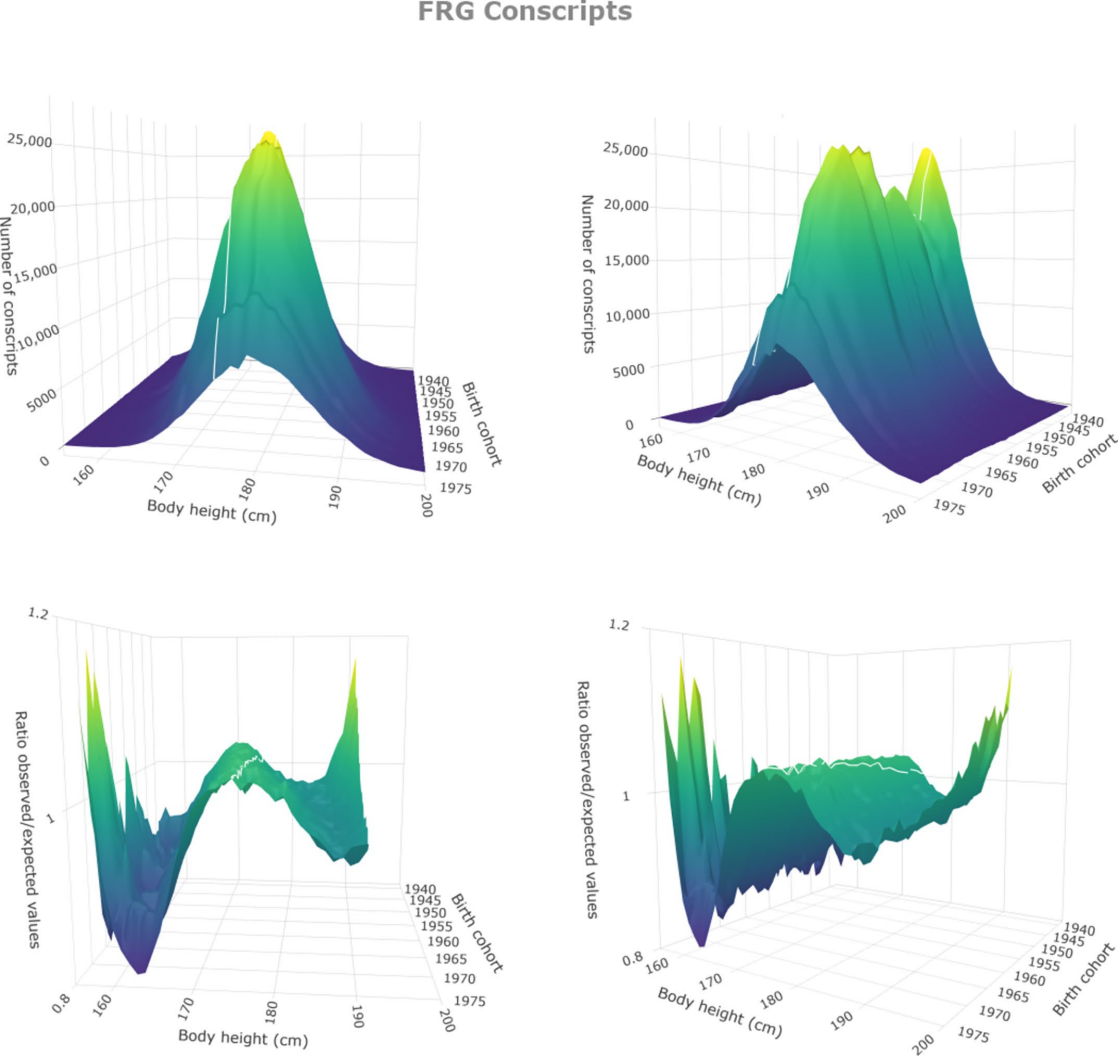
3D‐surface charts (two views) of the number of West German conscripts (born 1938–1975). Upper panel: Annual number of conscripts per centimeter class (frequency charts). Lower panel: o/e‐plot for annual height distributions. Mean height of all cohorts is indicated by a white line in order to better illustrate the trend in height over time. The irregularities at both sides of the o/e‐plots are due to small numbers.

**FIGURE 5:**
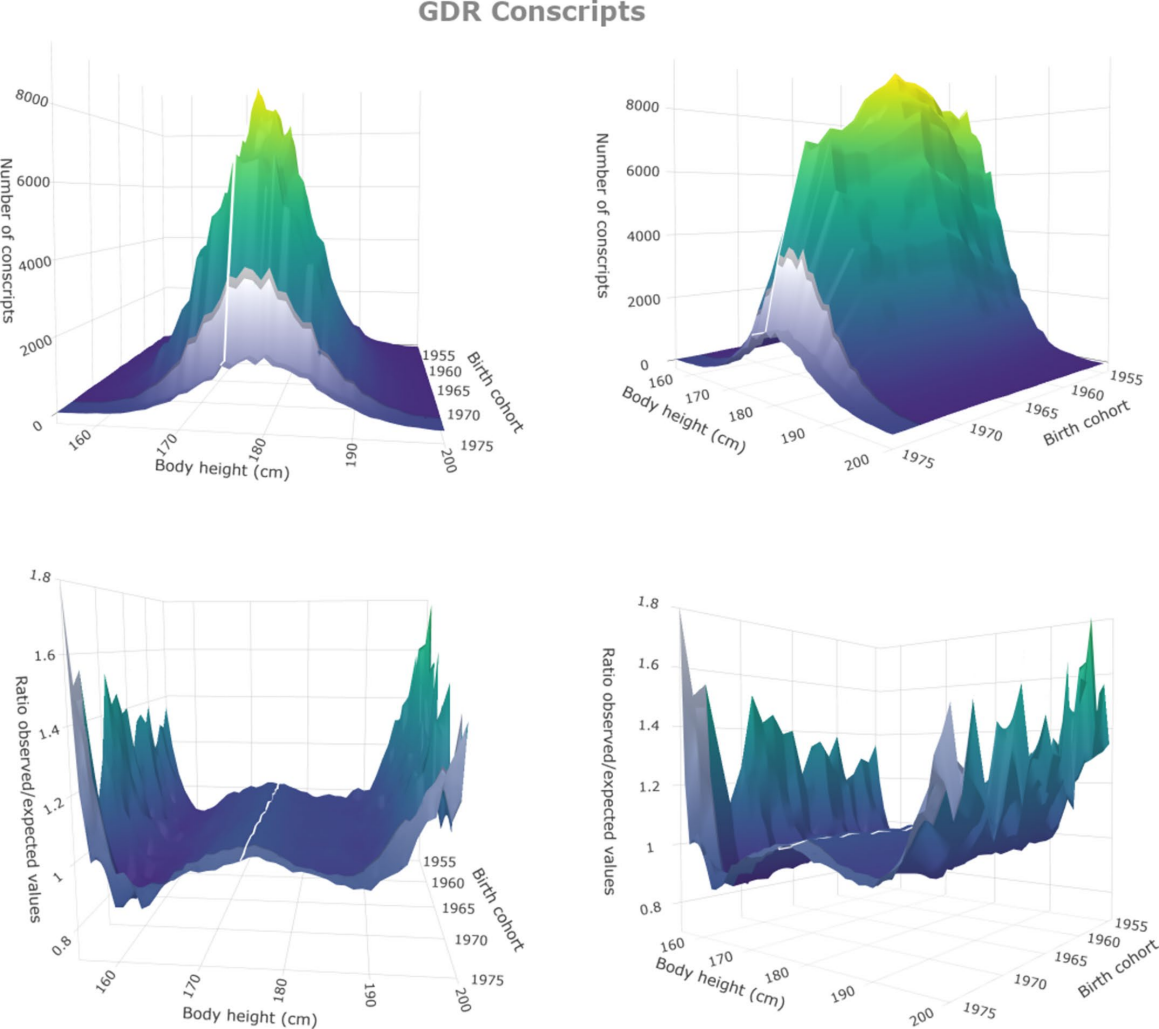
3D‐surface charts (two views) of the number of East German conscripts (born 1955–1975). Cohorts conscripted after the German reunification are colored gray. Upper panels: Annual number of conscripts per centimeter class (frequency charts). Lower panels: o/e‐plot for annual height distributions. Mean height of all cohorts is indicated by a white line in order to better illustrate the trend in height over time.

Figure 4 illustrates the height distributions of 12 458 211 West German conscripts [birth cohorts 1938–1975 (Institut für Wehrmedizinalstatistik und Berichtswesen, Remagen 1995)]. Figure 4 (upper panels) shows annual conscript numbers per centimeter class and confirms the well‐known Gaussian distribution of height (Boyd 1980). Figure 4 (lower panels) shows annual quotients of observed numbers divided by the expected Gaussian numbers of conscripts (o/e‐quotients). Slightly more than expected conscripts crowd around average height and fewer on either side of the average. Figure 5 illustrates conscript numbers per centimeter class and o/e‐quotients of 2 171 038 East German conscripts born 1955–1975 (Jäschke pers. commun.; Jäschke 1991), the last 3 cohorts being conscripted according to West German regulations (after the unification of Germany in 1990).

Figures 4 and 5 show height distributions that almost perfectly correspond to normal distribution without evidence of any changes in these distribution patterns over time. Even the substantial social, political, and economic turbulences after the German reunification lack corresponding turbulences in the height distributions.

### 4.1 The “transgenerational growth potential” and the Gedankenexperiment

Community effects restrict the full “transgenerational growth potential”. They serve as height attractor and narrow the variation in height. Mean body height (*μ*) and SD for height (σ) of the 1865 cohort was *μ*_1_ = 165.8 cm, *σ*_1_ = 6.51 cm, and in 1975, *μ*_2_ = 180.1 cm, and *σ*_2_ = 6.96 cm. Based on these values we calculated the “transgenerational growth potential” of German men with *μ* = 173 cm, and for reasons of simplicity, ignored the intermittent cohorts. As we considered the difference in SD between the 1865 and 1975 cohorts as negligible, we used an intermediate SD of 6.7 cm (i.e., a variance of 44.9 cm^2^) as an approximation of the within‐cohort height variation and determined the SD of the “transgenerational growth potential” by:

$$\begin{array}{c} \text{Variance}=\sigma^{2}+\frac{1}{4}{\left(\mu_{1}-\mu_{2}\right)}^{2}\\ =6.7\;{\mathrm{cm}}^{2}+\frac{1}{4}{\left(165.8\;\mathrm{cm}-180.1\;\mathrm{cm}\right)}^{2}\\ =44.9\;{\mathrm{cm}}^{2}+50.9\;{\mathrm{cm}}^{2}\\ =96.0\;{\mathrm{cm}}^{2} \end{array}$$

SD=9.8cm

**FIGURE 6:**
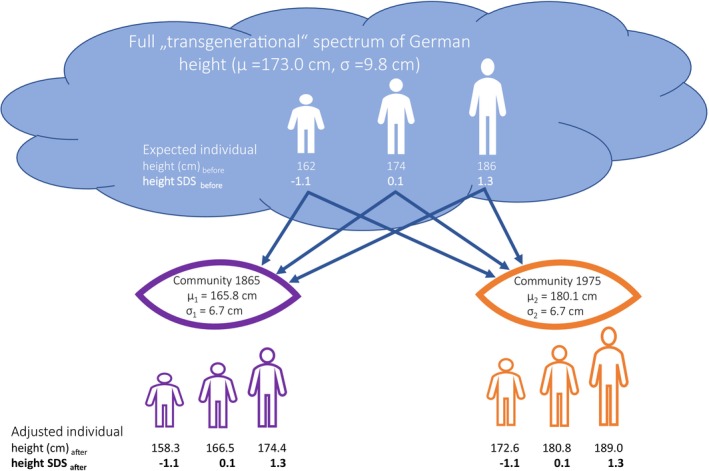
The Gedankenexperiment. We consider a case of virtual incarnation: three men (short, average, and tall) can decide whether to be born in the German Kaiserreich in 1865 or in the Federal Republic of Germany (FRG) in 1975. They belong to the “German gene pool” of all German men born between 1865 and 1975 and may encompass the full, albeit fictitious, spectrum of all historically possible heights (mean height *μ* = 173.0 cm, SD *σ* = 9.8 cm). Before incarnation, the short man may have a growth potential equivalent to a height SDS of −1.1 and we would expect him to achieve a final height of 162 cm within the spectrum of all possible heights; the average man may have a height SDS of +0.1, that is, we would expect him to reach 174 cm; and the tall man with height SDS of +1.3 would be expected to reach 186 cm. This however, is not the case. After entering the 1865 community (*μ*_1_ = 165.8 cm) and materializing their potential growth expectancy, the spectra of possible heights are shrunken: the short man will grow up to only 158.3 cm (height SDS = −1.1 in the 1865 community), the average man to 166.5 cm (height SDS = 0.1 in the 1865 community), and the tall man to 174.4 cm (height SDS = 1.3 in the 1865 community); whereas in the 1975 community (*μ*_2_ = 180.1 cm), the short man will reach 172.6 cm, the average man 180.8 cm, and the tall man 189 cm. The Gedankenexperiment considers that individual height retains its relative height position within the population (same height SDS), but adjusts to the local, resp. historical neighborhood. Entering a real‐life community reduces the “transgenerational growth potential” by 53%.

Starting from the, albeit fictitious, spectrum of all possible heights with a variance of 96.0 cm^2^, the Gedankenexperiment (Figure 6) shows that entering a real‐life community has reduced the initial height variance from 96.0 to just 44.9 cm^2^, that is, by 53%.

## 5 Discussion

Long‐term trends in height are linked to long‐term trends in economic, nutritional, and health conditions. This also applies to the German population and confirms previous observations (Case and Paxson 2008; Komlos 1994). Nevertheless, the apparently plausible notion that improvements in these conditions favor growth is deceptive. The glaring absence of tall people even in the most prosperous late German Kaiserreich, and the equally glaring absence of short people even among the poorest social classes of modern Germany rather support the impression that the link between height and living conditions may be fallacious.

Central and northern Europeans of the early and mid‐19th century were generally short. With 165 cm [Dutch, 1863 (Van Wieringen 1972)], 164 cm [Baden‐Württemberg, 1840–1864 (Jaeger et al. 2001)], 163.5 cm [Bavaria, 1875 (Jaeger et al. 2001)], and 163 cm (“the minimum height requirement to be a Prussian soldier was 5 feet (157 cm)). The 1811 survey of the Kantonisten of the line regiments gives the average height of the Prussian soldier as 163 cm” (Schmidt 2000)), average height of these people was close to the minimum ever observed among European people: the 15th century Norse Viking men in Greenland who had lived under worst conditions shortly before they disappeared, had an average height of 162 cm (Lynnerup 1998).

The impact of wealth on growth is known since the seminal work of Louis René Villermé [1782–1863 (Boyd 1980)] a French hygienist who formulated the idea that “the stature of man becomes higher, and growth is completed earlier, all things being equal, when the country is rich …”. Prosperity is associated with tall stature. The vision that body height reflects living conditions has meanwhile generally been accepted (Tanner 1986)—and was repeatedly confirmed since (Cole 2000; Duan et al. 2022; Fudvoye and Parent 2017; Hauspie et al. 1997; NCD‐RisC 2016): Men are tall when GDP is high (Case and Paxson 2008; Komlos 1994) and men are short when living in poverty. Yet, 19th Germans were not generally poor, nor malnourished, nor chronically ill. Quite in contrast, the German Kaiserreich enjoyed a booming industry that accounted for 15% of the global industrial production before the First World War (Scriba 2014). Most aristocrats and the leading urban upper class were exceptionally wealthy. The richest top 1% owned more than 40% of the total national wealth, the richest top 10% shared almost 80% of this wealth among themselves (Albers et al. 2020; Bartels and Hesse 2024). They enjoyed optimal living conditions including running tub water and waste water disposal that was supplied to most German cities and urban areas already at the end of the 19th century (Lange 2024).

Nutrition was abundant in those days. The inhabitants of the town of Leipzig consumed 73.6 kg of meat per year on average (Teuteberg 1971) which is more than the 52.0 kg/year that is consumed today (Bundesanstalt für Landwirtschaft und Ernährung 2024). The same applied for the consumption of milk and milk products which was similarly plentiful and available in almost all social classes. Fenton and Owen wrote (Fenton and Owen 1981): “As the following example of a medium‐sized German town shows, milk consumption rose noticeably at first only in the higher social classes”. Households in the city of Halle with a monthly income of 1200–1600 Reichsmark consumed 235 kg/year, households with a monthly income of 6000–7000 Reichsmark consumed 811 kg/year in 1909/1010 (Fenton and Owen 1981). The evidence of prosperity in the late 19th century German Empire is stunning. Yet, the people were short.

Even more arguments against the perception that prosperity is a necessary prerequisite for tall stature are found when considering modern people who live under socially and economically precarious circumstances: Contemporary poverty reports state that still in the 1990s, 10.1% of the German children lived in poverty. More than 50% of Hamburg's schoolchildren were considered in such poor nutritional condition that they had health problems or physical developmental delays. In Berlin, 16.2% of all children and young people under the age of 18 lived on social welfare at the end of the 20th century (DokZentrum ans Tageslicht 2015). But these children and adolescents grow well. The height distributions of German conscripts born 1938–1975 (Figures 4 and 5) lack evidence of substantial portions of short persons: Less than 3% of modern men (mean height 180.1 cm, SD 6.96 cm) are shorter than the 19th century average.

Nor can inadequate medical care be blamed for the short stature of the people of the German Empire. Spectacular improvements in public health took place at the end of the 19th and the early 20th centuries. Though infectious diseases were still common (Leven 1997), textbooks of pediatrics suggested that most children and adolescents easily survived these illnesses and appeared generally healthy (Gerhardt 1881).

Also, family size and education have changed. At the end of the 19th century, women gave birth to 4.7 children on average, whereas modern women tend to have less than 2 children. But households with no or few children were common at any time. Before 1900, 9% of the households had no children, and 9% had only one child. Twelve percent had two children (Statistisches Bundesamt Wiesbaden 1979). But the 9% of children from single child households and the 12% of children from the two‐children households did not reach modern height. Similar arguments may be put forward when considering education. The percentage of young men with university entrance qualifications had increased from 1.9% at the end of the 19th century to a maximum of 25.5% in 1990, and from 0.3% in 1921 to 27.6% in 1990 in women (Müller‐Benedict 2022). Academics are taller than workers, but the striking shift in the ratio between large and small families, and academics and non‐academics is not reflected in similar shifts in the distribution of height (lower panels in Figures 4 and 5).

The available evidence suggests rejecting the first two hypotheses—neither the SD nor the skewness of the body height distribution reflects the degree of social asymmetry. Height distributions closely follow an almost ideal symmetrical Gaussian shape with no apparent change over time. Gaussian distributions of height are not only found in modern populations but also in 19th and early 20th centuries Swiss (Staub), Scottish, Ligurian, Italian, Dutch (Hermanussen et al. 1995; Ripley 1897), and US American (Boyd 1980) men. The coefficients of variation for height, both in modern and feudal societies, equally range between 3.2% and 3.4% and do not reflect the numerical balance between affluent and poor people (Boyd 1980). Affluent and mostly well‐educated people tend to be relatively taller than poor and mostly low‐educated people within a given population, but height in absolute terms corresponds more to the average height of their contemporary neighbors, peers, and parents than to individual economic prosperity, nutritional status, health, and education.

When pooling height data of men born in different historic periods, the wealthy, healthy, well‐fed and educated people will no longer accumulate at the “tall end”, and the disadvantaged and poor at the “short end” of such distributions, but will spread across the whole range. We therefore accept the third hypothesis. Community effects serve as height attractor and restrict the variance of the “transgenerational growth potential” by at least half.

## 6 Height Is a Social Signal

We consider height as a social signal (Hermanussen and Scheffler 2016; Scheffler et al. 2021). Height includes the claim to a dominant position (Stulp et al. 2015). The interplay of social and neuroendocrine signaling (Hermanussen et al. 2022) determines the fine‐grain regulation of growth. Group membership sets strategic targets for height (Buston and Clutton‐Brock 2022) and restricts a potentially much greater range of height—the “transgenerational growth potential”—to a much narrower range around the height average of the group. Community‐related effects are particularly obvious in migrants (Bogin, Hermanussen, et al. 2018; Scheffler et al. 2021) when integrating into their host communities. Our Gedankenexperiment suggests that community effects limit the “transgenerational height potential” by at least half.

The present analysis may contribute to the understanding of “missing heritability”. Missing heritability describes the gap between heritability estimates from genotype data and heritability estimates from family data, and has been a source of debate for at least a decade (Young 2019). Since the family is the primary social group for a child and thus, the most important playground for community effects, we argue that “missing heritability” may simply reflect the magnitude of the community effect on height among close relatives.

Community effects on height have been described in Swiss (Hermanussen et al. 2014), Norwegian (Bents et al. 2017), Japanese (Bents et al. 2018), migrant (Bogin, Hermanussen, et al. 2018), and recently in Chinese people (Zhou et al. 2023). Community effects appear to protect from being “too tall” or “too short” within the relevant social group.

### 6.1 Limitations

The present analysis is based on published data. It refers to pooled information obtained at the level of the population, not at the level of the individual subject. We describe statistical associations that cannot be interpreted as causal relationships. When selecting the data, particular attention was paid to publications that preferably covered the complete time span of 110 years or at least a significant portion of this time span. Modern data sources of, for example, the World Bank or the WHO were therefore not used. Germany joined the International Bank for Reconstruction and Development (IBRD)—the World Bank—in 1952, and the International Development Association—the Bank's fund for the poorest countries—as late as in 1960 (https://www.worldbank.org/en/country/germany/overview). The WHO provides data on SES for Germany not before the 1990s (https://data.who.int/countries/276). Published data on longterm trends may be prone to inaccuracies. In particular, data that try to quantify social and economic inequality during periods of war and political unrest may be of particularly poor reliability. Therefore, we strongly recommend replicating our study in other European and non‐European countries.

Variation in height is associated with living conditions. The present study investigates this association within and not between birth cohorts, and it lacks information at the level of the single individual. The study does not contribute to the exploration of potential mechanisms that might explain long‐term trends in height at the level of the population (Cole 2000). This shall be the topic of another manuscript (Scheffler et al. 2025).

## 7 Conclusion

Body height reflects living conditions. Economic prosperity, nutrition, health, and education favor the physical growth of men. But these factors contribute to less than half of the ecological tolerance range of body height. The mean height of the social community is a strong attractor of individual height and appears to outweigh the effect of individual living conditions and to substantially narrow the transgenerational growth potential, to protect against being “too tall” or “too short”.

## Author Contributions

M.H. and C.S. conducted the study, C.A. contributed the statistics, M.H. wrote the first draft of the manuscript, C.S. gave crucial intellectual input.

## Ethics Statement

The authors have nothing to report.

## Conflicts of Interest

The authors declare no conflicts of interest.

## Supporting information


**APPENDIX S1.** Supporting information.

**TABLE S1.** Mean body height, standard deviation for height (height SD) and (after 1938) skewness for height of German men referred to year of birth.

## Data Availability

The data that support the findings of this study are available from the corresponding author upon reasonable request.
