# Supplementary material for: Avoiding “Too Tall” and “Too Short”: The Effect of the Community on the Regulation of Body Height
Source: Am J Hum Biol. 2025 Jun 12;37(6):e70085. doi: 10.1002/ajhb.70085 (PMC12159794; doi:10.1002/ajhb.70085)
Supplement: Supplementary file 1 — APPENDIX S1. Supporting information. [file AJHB-37-e70085-s002.docx]

**Avoiding “too tall” and “too short”: the effect of the community on the regulation of body height**

**Interactive 3D Plots of Figure 3 and 4**
